# Exploring the Role of Social Connection in Interventions With Military Veterans Diagnosed With Post-traumatic Stress Disorder: Systematic Narrative Review

**DOI:** 10.3389/fpsyg.2022.873885

**Published:** 2022-07-08

**Authors:** Richard D. Gettings, Jenna Kirtley, Gemma Wilson-Menzfeld, Gavin E. Oxburgh, Derek Farrell, Matthew D. Kiernan

**Affiliations:** ^1^Northern Hub for Veterans and Military Families Research, Northumbria University, Newcastle upon Tyne, United Kingdom; ^2^Department of Violence, Trauma and Criminology, Worcester University, Worcester, United Kingdom

**Keywords:** loneliness, mental health, military, Post-Traumatic Stress Disorder, psychosocial, social isolation, veteran

## Abstract

**Background:**

It has been identified that military veterans have distinct experiences of loneliness and social isolation and, when comparing this community to other client groups with a PTSD diagnosis, veterans respond less favorably to treatment. However, the link between PTSD and loneliness for veterans remains insufficiently researched and it is unclear if there are effective interventions tackling this distinct experience of loneliness.

**Aims:**

This systematic narrative review aimed to synthesize existing evidence incorporating elements of social connection, social isolation, and loneliness within interventions for military veterans with a diagnosis of PTSD, consequently aiming to examine the impact of such interventions upon this community.

**Methods:**

Six databases were searched, utilizing relevant search criteria, with no date restrictions. Articles were included if they involved intervention or treatment for military veterans with PTSD and considered elements of social connection, social isolation, and/or loneliness. The initial search returned 202 papers. After exclusions, removal of duplications, and a reference/citation search, 28 papers remained and were included in this review.

**Results:**

From the 28 studies, 11 directly addressed social isolation and two studies directly addressed loneliness. Six themes were generated: (i) rethinking the diagnosis of PTSD, (ii) holistic interventions, (iii) peer support, (iv) social reintegration, (v) empowerment through purpose and community, and (vi) building trust.

**Conclusions:**

A direct focus upon social reintegration and engagement, psychosocial functioning, building trust, peer support, group cohesiveness and empowerment through a sense of purpose and learning new skills may mitigate experiential loneliness and social isolation for veterans with PTSD. Future research and practice should further explore the needs of the PTSD-diagnosed veteran community, seek to explore and identify potential common routes toward the development of PTSD within this community and consider bespoke interventions for tackling loneliness.

## Introduction

The effects of trauma upon the function of the human brain have been known for millennia, reported diversely across ancient Greek, Roman and Hebrew literature, to name a few. Wherever global armies battled the effects of trauma upon the combatants was later reported and recorded for posterity. Under the guise of many different monikers, Shell Shock, Battle Fatigue, Soldier's Heart to name three, the consequences of combat upon cognitive function have been laid bare. These studies became more formalized in the nineteenth Century, culminating in Post-Traumatic Stress Disorder (PTSD) becoming a diagnosable condition in 1980, when the American Psychological Association included it in the Diagnostic and Statistical Manual of mental disorders (DSM), 3rd Edition (American Psychiatric Association, 1980). Eight percent of the general population will be affected by PTSD, at some stage in their life, figures which do not take into account the inevitable consequences of COVID upon many emergency service and public-facing occupations, a rate which is doubled for active duty service members and veterans (Judkins et al., 2020). In comparison, 17% of UK troops who were deployed in combat roles, during the Iraq and Afghanistan conflicts, later developed symptoms of PTSD; compared with 6% for those who were not deployed (Stevelink et al., 2018). This juxtaposition of PTSD prevalence rates is perhaps indicative of both the disparate nature of trauma faced by active-duty service personnel and the potentially incongruous, outdated and non-sufferer centric diagnostic, support and therapeutic processes instituted for the PTSD diagnosed, military and veteran, communities? (Iversen et al., 2009; Ginzburg et al., 2010).

PTSD is a prevalent and debilitating disorder amongst military personnel (serving and veterans) globally, having a long-term impact and creating a significant public health challenge (Steenkamp et al., 2015). It has been found to be associated with transition out of the military, taking hold of an individual potentially once they enter the void of psychological inactivity and lack of direction that faces many whom leave with little, or no, planning and preparation. It is, furthermore, associated with social exclusion and higher rates of deprivation (Karstoft et al., 2015; Sayer et al., 2015; Murphy et al., 2016). There is a certain latency with the development of PTSD, sometimes many years after transition (Marmar et al., 2015). Since 2001, in excess of 280,000 UK Service personnel have been deployed to combat zones in Iraq and Afghanistan (Carlson et al., 1998), and ~11–15,000 UK service personnel currently make the transition into civilian life each year (Ministry of Defence, 2021b). Advances in the treatment and diagnosis of PTSD have led to the differentiation between PTSD and Complex PTSD (C-PTSD) (Powers et al., 2017), whereby it is now recognized that early life traumatisation, prolonged and multiple traumas, deep-seated and unresolved symptoms may prove to be the catalysts for the complex derivative of the disorder; significantly diagnosed within the military and veteran communities due, potentially, to the recruitment dynamic of those who join the military and the idiosyncratic nature of training, experience and culture (Wilson et al., 2018). Parallel advances in the identification, and detailed examination, of loneliness and its psychological correlates, such as feelings of shame and guilt, difficulty controlling emotions, dissociation, feeling cut off from family and friends and risky behavior (Walton et al., 1991) have led to an increasing awareness of the true experience of loneliness and social isolation. These symptoms are not captured in the existing PTSD diagnostic criteria in either the International Classification of Diseases (ICD), 11th edition or the DSM 5 (American Psychiatric Association, 2013; World Health Organization, 2020) (see Table 1). Amongst this panoply of additionally recognized symptoms and consequences are experiences that can be associated with loneliness and social isolation.

**Table 1:** Symptom capture and limitations on existing PTSD criterion.

**Symptoms captured by existing PTSD criterion (ICD-11/DSM 5)**	**Symptoms not captured by existing PTSD criterion (ICD-11/DSM 5)**
° Fear ° Re-experiencing ° Avoidance behavior ° Hypervigilance ° Horror ° Helplessness ° Challenge to physical integrity ° Psychogenic amnesia ° Reduced affect ° Dissociation ° Anger ° Impact on functioning ° Self-blame ° Self-destructiveness ° Alterations in world view	° Depression ° Guilt ° Shame ° Psychosexual difficulties ° Betrayal ° Stigmatization ° Self-medicating activity ° Increased vulnerability to re-traumatisation

Loneliness is a subjective social and emotional experience, often traditionally characterized as the difference between the social relationships individuals actually have and those that they aspire to having (Walton et al., 1991). Conversely, social isolation is an objective experience which considers the integration of the individual into their social environment, the frequency of their social interactions and their integration within social networks (Cacioppo et al., 2006). Research shows that loneliness and social isolation are linked to poor physical health and wellbeing, including high blood pressure, cognitive decline, depression, and mortality (Cacioppo et al., 2006; Holt-Lunstad et al., 2010; Steptoe et al., 2013) and are global issues affecting individuals of all ages.

Evidence demonstrates the unique experiences and needs of military veterans in terms of social isolation and loneliness (Wilson et al., 2018). These unique experiences stem from both intrinsic and extrinsic factors related to military life, such as military-related trauma and PTSD. Transition, and losing touch with comrades was another factor which influenced experiences of loneliness and social isolation (Wilson et al., 2018). Further recent research from two of the largest UK military charities, Royal British Legion and the Soldiers, Sailors, Airmen and Families Association (Royal British Legion, 2014; SSAFA, 2017) indicates that 41% of veterans surveyed (over 2,000 veterans, aged 18–64, participated) had personally experienced loneliness or social isolation and 27% had experienced suicidal ideation, since transitioning from the military to civilian life.

Shepherd et al. (2020) highlight the many challenges of transition and throw light upon cultural and structural differences between the military and civilian communities which facilitate and aggravate these difficulties. A recent US military family lifestyle survey (Sonethavillay et al., 2018) reported that 47% of veteran families had a difficult or very difficult transition experience due to loss of connection and purpose, stress, depression and suicidal thoughts. It is argued that these were exacerbated by frequent relocations and disruption of the established friendship bonds and community links (Stapleton, 2018). Woodward and Jenkings (2011) encapsulated the term “fictive kinship” to describe the practice of considering the military as “family”. The potential loss of this military family becomes a catalyst for “experiential isolation”, the truly unique and extraordinary psychological circumstances that veterans find themselves in; suddenly unable to bond psychologically with members of their family and friends and being unable to share a common empathy or moral compass (Stein and Tuval-Mashiach, 2014, 2015). Previously accepted and established value-system goalposts are suddenly moved, and ethical and social signposts are taken away; leaving the transitioning veteran isolated and estranged.

It is argued that a comorbidity exists between loneliness and PTSD symptomology. Ypsilanti et al. (2020) concluded that self-disgust and loneliness simultaneously predict PTSD symptomology, and these two measures play a cooperative role in predicting anxiety and depression. Research affirms that loneliness and social isolation are uniquely linked to PTSD symptomology *via* the catalyst of Combat Stress Reaction (Solomon et al., 1986, 2013); idiosyncratic trigger points that relate to military culture, lived experience and the distinctive pressures exerted by transitioning from the military to civilian life (Keats, 2010). These issues are known to be aggravated by mental health stigmatization, denial and avoidance within the military and veteran communities (Rozanova et al., 2015). A comorbidity potentially exists between PTSD, loneliness, and suicide (Pietrzak et al., 2017; Yael and Yager, 2019). A systematic review and meta-analysis of the link between loneliness and suicidal ideation concluded that loneliness was indeed a significant predictor of suicidal ideation in select communities (McClelland et al., 2020). However, more focused research is now required to gain a better understanding of the unique veteran experience of loneliness, and to subsequently aid the design of interventions aimed at reducing loneliness, social isolation and the consequent rates of suicide and suicidal ideation amongst this community.

### Aims of Current Study

This systematic narrative review aims to synthesize existing evidence incorporating elements of social connection, social isolation, and loneliness within interventions for military veterans with a diagnosis of PTSD, consequently aiming to examine the impact of such interventions upon this community.

## Methods

A systematic narrative literature review was conducted. Ethical approval was not required due to it being a review only. Six identified databases were searched (see Table 2; Popay et al., 2012; Snilstveit et al., 2012). Inclusion/exclusion criteria were applied that the accepted studies must involve intervention or treatment for military veterans with PTSD and consider elements of social connection, social isolation, and/or loneliness. Papers must have been written in English and, could not be review papers (see Table 2).

**Table 2:** Systematic search strategy.

Source	ASSIA ETHOS PsycARTICLES Science direct freedom collection Scopus Web of science
Search field	Title and abstract
Exclusion	Non-English language Literature reviews
Year of publication	All years
Search Terms	(Veteran OR ex-servic* OR ex-forc* OR ex-militar*) AND (social isolation OR lonel*) AND (post traumatic stress OR post traumatic stress disorder OR PTSD OR trauma*)

**Figure 1:**
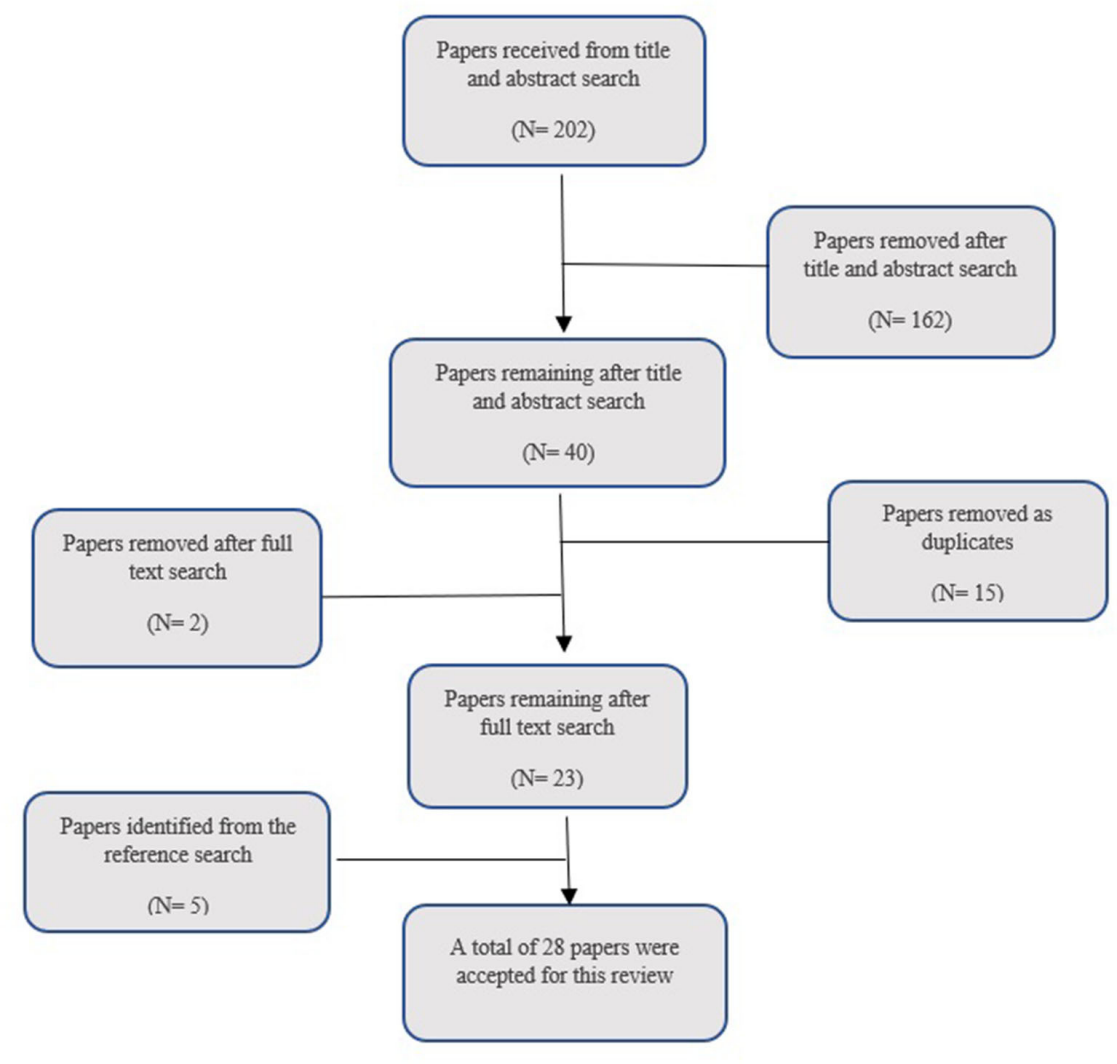
Search strategy used within the systematic search (Moher et al., 2009).

A total of 202 articles were identified from the title and abstract search (Figure 1; Moher et al., 2009). However, 162 were removed as they did not meet the inclusion criteria, i.e., they were not written in English, did not include any aspect of social connection within the intervention, or did not include military veterans diagnosed with PTSD. Fifteen papers were duplicates and thus, were removed. From the 25 remaining studies, a full-text search was conducted, and two further papers were excluded as it was found that they also did not fulfill the inclusion criteria. A reference and citation search was carried out on all included papers, and this resulted in five further papers being included. A total of 28 papers were included in this review (see Supplementary Material).

Data analysis was undertaken, using reflexive, deductive thematic synthesis (Braun and Clarke, 2006, 2014, 2019) to generate themes. Specifically, the six stages of Thematic Analysis were followed: generating initial codes; searching for themes; reviewing themes; defining and naming themes; and producing the report (Braun and Clarke, 2006). A collaborative approach to coding and data analysis was taken by members of the research team. Initial codes and were discussed between the research team and final themes/sub-themes were generated based on this collective analysis. Given that this is a review into a novel, and potentially pioneering, aspect of military veteran PTSD prognosis and support, it was decided not to utilize any quality assessment tool, such as CASP for this systematic literature review as it would have been counter-productive to exclude any of the identified papers based upon their conceived quality of contribution.

## Results

Six main themes were generated reflecting the findings of the 28 identified studies: (i) rethinking the diagnosis of PTSD; (ii) holistic interventions; (iii) peer support; (iv) social reintegration; (v) empowerment through purpose and community, and; (vi) building trust.

The age of veterans differed between the studies, most being non-specific with regards to age. Seventeen studies included veterans of all ages (Jones et al., 2000; Bensimon et al., 2008, 2012; Holliday et al., 2015; Azevedo et al., 2016; Beidel et al., 2017; Bergen-Cico et al., 2018; Crowe et al., 2018; Johnson et al., 2018; Lobban and Murphy, 2018, 2020; Pezzin et al., 2018; Weiss et al., 2018; Bolman, 2019; McLaughlin and Hamilton, 2019; Galsgaard and Eskelund, 2020; Bauer et al., 2021), whereas four studies were age-specific by virtue of the criteria that they sought veterans from the Vietnam War (1961-75) (Obenchain and Silver, 1991; Ragsdale et al., 1996; Johnson et al., 2004; Otter and Currie, 2004) or Post-9/11/Iraq and Afghanistan veterans (Beidel et al., 2016; Lawrence et al., 2017, 2019; Matthieu et al., 2017; Cushing et al., 2018). Three studies focused solely on female veterans, with two of these relating to rural female veterans who had suffered military sexual trauma (Azevedo et al., 2016; Weiss et al., 2018), and the other relating to a single case study (Trahan et al., 2016).

Eight studies utilized animal-focused interventions (Nevins et al., 2013; Trahan et al., 2016; Bergen-Cico et al., 2018; Crowe et al., 2018; Johnson et al., 2018; Bolman, 2019; McLaughlin and Hamilton, 2019; Galsgaard and Eskelund, 2020). Three studies investigated the efficacy of music related interventions (Bensimon et al., 2008, 2012; Pezzin et al., 2018). One study focused upon yoga as an intervention (Cushing et al., 2018), one upon an adventure-activity intervention (Ragsdale et al., 1996), whilst three studies examined the power of civic service to ameliorate PTSD symptomology (Lawrence et al., 2017, 2019; Matthieu et al., 2017). One study focused upon military museums and art therapy (Lobban and Murphy, 2020), another solely upon art therapy (Lobban and Murphy, 2018), whilst another investigated the efficacy of virtual reality exposure as a suitable medium for PTSD intervention (Beidel et al., 2016). One study involved exercise to mediate PTSD symptomology (Otter and Currie, 2004), whilst one (Johnson et al., 2004) sought to incorporate a veteran's family into the whole treatment and support process.

The vast majority of studies (*n* = 21) were carried out in the USA (Obenchain and Silver, 1991; Ragsdale et al., 1996; Jones et al., 2000; Johnson et al., 2004, 2018; Holliday et al., 2015; Azevedo et al., 2016; Beidel et al., 2016, 2017; Trahan et al., 2016; Lawrence et al., 2017, 2019; Matthieu et al., 2017; Bergen-Cico et al., 2018; Crowe et al., 2018; Cushing et al., 2018; Pezzin et al., 2018; Weiss et al., 2018; Bolman, 2019; Bauer et al., 2021) whilst two were conducted in Israel (Bensimon et al., 2008, 2012), two in the UK (Lobban and Murphy, 2018, 2020), one in Denmark (Galsgaard and Eskelund, 2020) and two in Australia (Otter and Currie, 2004; McLaughlin and Hamilton, 2019).

Seven studies used a mixed-methods approach (Johnson et al., 2004; Bensimon et al., 2008, 2012; Beidel et al., 2016, 2017; Bergen-Cico et al., 2018; Lobban and Murphy, 2020), 12 studies used a quantitative approach (Ragsdale et al., 1996; Holliday et al., 2015; Trahan et al., 2016; Lawrence et al., 2017, 2019; Matthieu et al., 2017; Johnson et al., 2018; Lobban and Murphy, 2018; Pezzin et al., 2018; Weiss et al., 2018; Bauer et al., 2021) and nine used a qualitative approach (Obenchain and Silver, 1991; Jones et al., 2000; Otter and Currie, 2004; Azevedo et al., 2016; Crowe et al., 2018; Cushing et al., 2018; Bolman, 2019; McLaughlin and Hamilton, 2019; Galsgaard and Eskelund, 2020). Within each broad methodological approach a variety of methods were employed; 11 studies employed focus groups or a group-centric approach (Obenchain and Silver, 1991; Ragsdale et al., 1996; Jones et al., 2000; Johnson et al., 2004; Otter and Currie, 2004; Bensimon et al., 2008, 2012; Crowe et al., 2018; Lobban and Murphy, 2018, 2020; Galsgaard and Eskelund, 2020), eight used semi-structured interviews (Johnson et al., 2004; Bensimon et al., 2008, 2012; Beidel et al., 2016, 2017; Crowe et al., 2018; Cushing et al., 2018; Galsgaard and Eskelund, 2020) and 16 utilized questionnaires (Holliday et al., 2015; Azevedo et al., 2016; Beidel et al., 2016, 2017; Trahan et al., 2016; Lawrence et al., 2017, 2019; Matthieu et al., 2017; Johnson et al., 2018; Lobban and Murphy, 2018, 2020; Weiss et al., 2018; McLaughlin and Hamilton, 2019; Bauer et al., 2021). Two studies were quasi-experimental (Ragsdale et al., 1996; Bergen-Cico et al., 2018), two studies utilized a randomized trial (Johnson et al., 2018; Pezzin et al., 2018) and two a controlled trial (Beidel et al., 2016, 2017). Five studies were pilot analyses (Bensimon et al., 2012; Beidel et al., 2017; Pezzin et al., 2018; Weiss et al., 2018; Galsgaard and Eskelund, 2020).

Of the studies using questionnaires, six specifically measured loneliness, using either the SELSA (Johnson et al., 2018) or UCLA loneliness self-report scales (Ragsdale et al., 1996; Lawrence et al., 2017, 2019; Matthieu et al., 2017; Lobban and Murphy, 2020).

### Main Theme 1: Rethinking the Diagnosis of PTSD

A central banner that emanates from almost all of the identified studies, other than those focused on talking therapies such as Cognitive Processing Therapy (CPT; Holliday et al., 2015) and Cognitive Behavior Therapy (CBT; Trahan et al., 2016), is an acceptance that the current parameters of PTSD diagnoses and treatment are, perhaps, too narrow and restrictive. The strength of the interventions explored here emanates from their recognition of the need to tackle loneliness and social isolation. The diagnosis and treatment of PTSD has, potentially, been viewed in too reductionist a fashion, relying too heavily on the traditional views and approaches; rather than seeing the existential and moral dimensions of treating the whole person holistically (Walton et al., 1991; Iversen et al., 2009; Ginzburg et al., 2010; McFarlane, 2014).

Walton et al. (1991) and Cacioppo et al. (2006), assisted by Wilson et al. (2018) who provide the bespoke nature of military and veteran community, assist moving the dialogue, relating to what the true inherent ingredients of loneliness and social isolation really are, to where it needs to be to be current and relevant for military and veteran PTSD. Such red flags as depression, guilt, shame, psychosexual difficulties, betrayal, stigmatization, self-medication and addiction and increased vulnerability to re-traumatisation all contribute to the destructive cocktail that manifests in the loneliness and social isolation of those living with a PTSD diagnosis in these communities (Walton et al., 1991; Van Ommeren et al., 2002; Iversen et al., 2009; Ginzburg et al., 2010; Palic and Elklit, 2011). Any fit for purpose system, therefore, which seeks to diagnose, signpost and support these communities must acknowledge and accommodate these catalysts.

### Main Theme 2: Holistic Interventions

Studies within this review identify the use of animals (Matthieu et al., 2017; Bergen-Cico et al., 2018; Crowe et al., 2018; Johnson et al., 2018; Bolman, 2019; McLaughlin and Hamilton, 2019; Galsgaard and Eskelund, 2020), music (Bensimon et al., 2008, 2012; Pezzin et al., 2018), art and museums (Lobban and Murphy, 2018, 2020) and adventure training (Ragsdale et al., 1996) as holistic interventions, which seek to offer the PTSD diagnosed veteran meaningful engagement, social connections, and a sense of purpose, thus ameliorating the negative mindset maintained by loneliness and social isolation; holistic in as much as they offer a treatment of mind and body as a whole, *via* the conduit of addressing the often ignored social, emotional and personal catalysts. Four studies found that dogs offered a non-judgmental, unconditional, support and buffer, facilitating responsibility and a sense of purpose (Bergen-Cico et al., 2018; Crowe et al., 2018; McLaughlin and Hamilton, 2019; Galsgaard and Eskelund, 2020). The study that focused on caring for a traumatized parrot fostered a sense of “becoming well together” and mutually shared suffering and empathy (Bolman, 2019). The studies that explored horse riding therapy found that the veterans were able to build mastery, improve mindfulness skills, and were able to connect with the animal (Nevins et al., 2013; Johnson et al., 2018). These animal-centric studies assessed outcomes *via* a combination of PTSD, loneliness, and social isolation metrics and group interviews. One study (Obenchain and Silver, 1991) provided Vietnam veterans an opportunity to address social isolation and “alienation” *via* the affirmation provided through a “Welcoming Home” ceremony, which aided societal participation and reintegration. The efficacy of these interventions appears to be that they focus on improving the overall wellbeing of the veteran, considering the various social and personal contributors to their difficulties, rather than focusing on PTSD symptomology in isolation.

### Main Theme 3: Peer Support

A dominant theme running through the identified studies was the power of peer support in fostering a suitable environment for an intervention to be effective; a significant number adopting a group focused delivery approach (Obenchain and Silver, 1991; Ragsdale et al., 1996; Jones et al., 2000; Johnson et al., 2004; Otter and Currie, 2004; Bensimon et al., 2008, 2012; Crowe et al., 2018; Lobban and Murphy, 2018, 2020; Galsgaard and Eskelund, 2020), and many others taking advantage of this group dynamic indirectly through the creation of a “team atmosphere”. The worth of creating an environment which is accepting, normalizing, and non-judgmental for PTSD diagnosed veterans is clear; an atmosphere of shared understanding empowering the healing process (Obenchain and Silver, 1991; Lobban and Murphy, 2018, 2020). This approach acknowledges and utilizes a strength of the military and veteran communities; its sense of brother/sisterhood, its “fictive kinship” (Woodward and Jenkings, 2011), that helps veterans re-engage and re-motivate each other and take a lead in their own respective recovery journeys.

### Main Theme 4: Social Reintegration

Through the mitigation of a veteran's estrangement from their community, loved ones, friends and family, it is possible to cultivate an environment where mindfulness, self-awareness and motivation is developed; in contrast to an individual treatment that focuses solely on tackling PTSD symptoms. Within the veteran community, difficulties with social interaction predict lower reductions in PTSD symptomology after treatment interventions such as CPT, CBT and pharmacology (Holliday et al., 2015; Trahan et al., 2016). However, the holistic interventions, which address the disenfranchisement and estrangement experienced by a transitioning veteran and their family (Obenchain and Silver, 1991; Ragsdale et al., 1996; Jones et al., 2000; Johnson et al., 2004, 2018; Otter and Currie, 2004; Bensimon et al., 2008, 2012; Azevedo et al., 2016; Trahan et al., 2016; Lawrence et al., 2017, 2019; Matthieu et al., 2017; Bergen-Cico et al., 2018; Crowe et al., 2018; Cushing et al., 2018; Lobban and Murphy, 2018, 2020; Pezzin et al., 2018; Weiss et al., 2018; Bolman, 2019; McLaughlin and Hamilton, 2019; Galsgaard and Eskelund, 2020; Bauer et al., 2021), generally show either a direct reduction of PTSD symptomology (Ragsdale et al., 1996; Nevins et al., 2013; Beidel et al., 2016, 2017; Trahan et al., 2016; Lawrence et al., 2017, 2019; Matthieu et al., 2017; Bergen-Cico et al., 2018; Johnson et al., 2018; Lobban and Murphy, 2018; Pezzin et al., 2018; Weiss et al., 2018; Galsgaard and Eskelund, 2020; Bauer et al., 2021) or an indirect reduction of such *via* mitigation of social estrangement and isolation (Obenchain and Silver, 1991; Johnson et al., 2004, 2018; Nevins et al., 2013; Holliday et al., 2015; Beidel et al., 2017; Matthieu et al., 2017; Bergen-Cico et al., 2018; Lobban and Murphy, 2018; Weiss et al., 2018; Bauer et al., 2021). More inclusive interventions, which seek to target the panoply of social integration and quality of life issues, would appear to address the whole lifestyle and culture of symptomology that is endemic within veteran PTSD; taking account of the unique community that the veteran has emerged from, providing a bespoke alternative to focused talking therapy or medication, allowing the veteran to take control over their prognosis and empowering their drive to recovery. Some studies (Bensimon et al., 2008, 2012; Pezzin et al., 2018) targeted group cohesion, togetherness, and connectedness, empowering veterans to gain a sense of control and esteem through mastery of a new skill. Retention and attrition rates are improved through the distraction and mindfulness facilitated by learning a musical instrument (Pezzin et al., 2018). The art and museum interventions (Lobban and Murphy, 2018, 2020) developed a sense of belonging, group bonding and self-efficacy through targeting experiential avoidance and assessing this progress through workshops and interviews. “Sanctuary Trauma”, the internal conflict within veterans relating to the apparent deficiency of their home environment, once they transition from the military, was addressed by the application of a “Welcome Home” ceremony to Vietnam veterans in one of the studies (Obenchain and Silver, 1991); aiming to focus upon their sense of social isolation and “alienation” through a process of re-affirmation, reintegration, and societal participation. It is, perhaps, surprising that only one study (Johnson et al., 2004) sought to involve the family of diagnosed veterans within the treatment and support process. Family-centric preventive interventions have habitually manifested a high efficacy for promoting positive outcomes with the PTSD diagnosed veteran and supporting family unit, and for encouraging consistent engagement, retention and focus upon the recovery journey (Lester et al., 2016).

### Main Theme 5: Empowerment Through Purpose and Community

Empowerment of PTSD diagnosed veterans through the facilitation of hope, purpose, challenge, direction and community (Obenchain and Silver, 1991; Beidel et al., 2016, 2017; Lawrence et al., 2017; Cushing et al., 2018; Lobban and Murphy, 2018; Weiss et al., 2018; Bauer et al., 2021) creates motivation, self-efficacy and opportunity for the veteran, *via* skill-building interventions, increasing their employability (Azevedo et al., 2016; Weiss et al., 2018; Bauer et al., 2021) and enabling their enrolment in voluntary schemes that contribute to the good of the community; promoting pride, improved self-esteem, increased confidence, courage and resilience (Lawrence et al., 2017, 2019; Matthieu et al., 2017).

### Main Theme 6: Building Trust

None of the above-mentioned themes would have any efficacy or power without the full investment, engagement and commitment of the PTSD diagnosed veterans who need support, guidance and signposting, many at the nadir of their respective journeys. Levels of veteran attrition and disengagement are high for the traditional PTSD therapies that they are directed toward (Haveman-Gould and Newman, 2018), this being contingent upon endemic levels of distrust amongst this community regarding the relevance and appropriacy of these measures and the disconnect they see between themselves and the “white coat” experts who tell them what is best for themselves. Effective interventions must, therefore, be able to empower and facilitate high levels of trust amongst the PTSD diagnosed veteran community. Holistic therapies succeed in fostering the required levels of trust by placing the veteran more centrally in the process, empowering them to feel as if they driving their own recovery, and not merely a passenger in someone else's vehicle (Obenchain and Silver, 1991; Johnson et al., 2004; Azevedo et al., 2016; Beidel et al., 2017; Lawrence et al., 2017, 2019; Matthieu et al., 2017; Weiss et al., 2018; Bauer et al., 2021).

## Discussion

The purpose of this review was to explore the effectiveness of interventions for tackling loneliness and social isolation in PTSD-diagnosed military veterans. Six themes were generated: (i) rethinking PTSD as a diagnosis; (ii) holistic intervention; (iii) peer support; (iv) social integration; (v) empowerment through purpose and community; and (vi) building trust. The papers highlighted that holistic interventions which can mitigate experiential loneliness and social isolation for veterans with PTSD include the following characteristics: a direct focus upon social reintegration and engagement, psychosocial functioning, building trust, peer support, group cohesiveness, empowerment through a sense of purpose and learning new skills. Peer and group-oriented holistic interventions were able to effectively target loneliness, and social isolation through improvements in the veterans' social and community engagement, self- efficacy, self-purpose, and through instilling hope and direction.

This review highlights the importance of socially reintegrating a PTSD-diagnosed veteran back within their community and with their loved ones. This social reintegration is a prerequisite of an effective treatment and a positive recovery journey; fostering growth and engagement through the conduit of group cohesion, togetherness, and connectedness. Linking the veteran back in with their vital support structures and, most importantly, empowering them to be able to communicate openly and honestly with that network, is paramount (Obenchain and Silver, 1991; Azevedo et al., 2016; Lawrence et al., 2017, 2019; Matthieu et al., 2017; Weiss et al., 2018; Bauer et al., 2021). As the studies suggest, the issues of alienation and stigmatization can be countered effectively, but also subtly (Ragsdale et al., 1996; Bensimon et al., 2008, 2012; Bergen-Cico et al., 2018; Crowe et al., 2018; Cushing et al., 2018; Johnson et al., 2018; Lobban and Murphy, 2018, 2020; Pezzin et al., 2018; Bolman, 2019; McLaughlin and Hamilton, 2019; Galsgaard and Eskelund, 2020). The panoply of issues, logistical and psychological, encountered by a veteran transitioning from the military to civilian life need to have been effectively identified, pre-empted and addressed (Obenchain and Silver, 1991; Johnson et al., 2004; Azevedo et al., 2016; Lawrence et al., 2017, 2019; Matthieu et al., 2017; Weiss et al., 2018; Bauer et al., 2021) as is highlighted in recent research and policy focused on understanding the needs of, and supporting, veterans through transition (Keats, 2010; Royal British Legion, 2014; SSAFA, 2017; Cooper et al., 2018; HM Government, 2018; Sonethavillay et al., 2018; National Health Service England, 2019; Shepherd et al., 2020; Ministry of Defence, 2021a).

A significant number of the studies also highlight the importance of trust (Obenchain and Silver, 1991; Johnson et al., 2004; Azevedo et al., 2016; Beidel et al., 2017; Lawrence et al., 2017, 2019; Matthieu et al., 2017; Weiss et al., 2018; Bauer et al., 2021). Engagement with any support and recovery mechanism is contingent upon the veteran trusting the process; trust of those involved in the process, trusting the agenda, and trusting the aspirations of the process. Trust takes time to build, especially within the PTSD-diagnosed veteran community which has become alienated and estranged from both their natural environment, the military community which it has now left, and from their new civilian environment, which it fails to identify or reconcile with. Awareness of the needs for interventions to “culturally adapt” to uniquely homogenous communities is evolving, even within the military and veteran worlds, but lessons learned need to be consolidated and cultivated further (Whealin et al., 2017). Transparency, openness and listening sincerely to the needs, aspirations and fears of the veteran community are key. As highlighted by recent UK Government strategies and NHS trusts, effective intervention should accommodate these needs, and seek to build the necessary trust (Whealin et al., 2017; HM Government, 2018; National Health Service England, 2019; Ministry of Defence, 2021a). Active involvement of the PTSD-diagnosed veteran community within care planning and transition (HM Government, 2018; National Health Service England, 2019) and within the design and construction stages of interventions through research (Bortoli, 2021) are some ways of effectively achieving this.

The power of peer-centered support and mutual understanding provides a sense of non-judgmental acceptance and normalization (Obenchain and Silver, 1991; Ragsdale et al., 1996; Jones et al., 2000; Johnson et al., 2004; Otter and Currie, 2004; Bensimon et al., 2008, 2012; Crowe et al., 2018; Lobban and Murphy, 2018, 2020; Galsgaard and Eskelund, 2020) and supports the mitigation of loneliness within PTSD. This camaraderie is especially important at the time of transition, when a serving member of the armed forces becomes a veteran. This is due to the potential sudden loss of social connectedness and intense bonds of friendship that they had during military service (Cooper et al., 2018; National Health Service England, 2019; Ministry of Defence, 2021a). A PTSD diagnosed veteran is more so able to normalize, accept, and consequently manage, their symptomology once they are around their similarly diagnosed comrades (Obenchain and Silver, 1991; Ragsdale et al., 1996; Jones et al., 2000; Johnson et al., 2004; Otter and Currie, 2004; Bensimon et al., 2008, 2012; Crowe et al., 2018; Lobban and Murphy, 2018, 2020; Galsgaard and Eskelund, 2020). The commonality of suffering and common language of military/veteran PTSD, built upon the forged “fictive kinship” (Woodward and Jenkings, 2011), is a powerful conduit to achieve these monumental and necessary steps toward normalization, acceptance, and management; and the fostering of the necessary purpose, meaning, and hope (Obenchain and Silver, 1991; Beidel et al., 2016, 2017; Lawrence et al., 2017; Cushing et al., 2018; Lobban and Murphy, 2018; Weiss et al., 2018; Bauer et al., 2021). In this manner, peer support becomes a complimentary mechanism for increasing treatment engagement and reducing negativity, pessimism, and dropout from veterans who are receiving ongoing support, through investing them in a process in which they feel central (Hundt et al., 2015).

Symptom-centric interventions tend to focus upon mental health professionals “doing” interventions to the veterans, but research indicates that patients are likely to drop out of treatment if they do not receive what it is they feel they need (Veeninga and Hafkenscheid, 2004). The findings of this review suggest that holistic, non-symptom focused, interventions have a productive role to play in the mitigation of veteran PTSD symptomology, its treatment and support, especially perhaps during the period of transition (Obenchain and Silver, 1991; Ragsdale et al., 1996; Jones et al., 2000; Johnson et al., 2004, 2018; Otter and Currie, 2004; Bensimon et al., 2008, 2012; Azevedo et al., 2016; Trahan et al., 2016; Lawrence et al., 2017, 2019; Matthieu et al., 2017; Bergen-Cico et al., 2018; Crowe et al., 2018; Cushing et al., 2018; Lobban and Murphy, 2018, 2020; Pezzin et al., 2018; Weiss et al., 2018; Bolman, 2019; McLaughlin and Hamilton, 2019; Galsgaard and Eskelund, 2020; Bauer et al., 2021); perhaps working in collaboration with, and sometimes working in place of, more traditional medical, psychological and pharmacological approaches. These interventions appear to be effective because they target the overall wellbeing of the veteran, rather than focusing on PTSD symptomology in isolation. The studies identified in the review broach this issue by fostering an atmosphere of trust, normalization, and mutual endeavor, which may facilitate the PTSD diagnosed veterans' investment in the whole process of “therapy” and taking personal ownership of their recovery pathways. Recent research developments, regarding holistic, non-trauma focused, interventions, coupled with the raising of awareness levels of what it is to be a transitioning veteran (Hundt et al., 2015; Cooper et al., 2018; HM Government, 2018; Wilson et al., 2018, 2020; McGill et al., 2019; National Health Service England, 2019; Ministry of Defence, 2021a), should include a focus on the effective mitigation of the loneliness and social isolation elements of veteran PTSD symptomology, in accordance with the Defense holistic transition policy (Ministry of Defence, 2021a).

The implementation of a holistic and personalized approach, and empowering veterans to be involved in their recovery, was a running theme within the studies included in the review. This is in line with the UK Government and the Ministry of Defense strategy, which addresses the needs, concerns and aspirations of the military and veteran communities with regards to their mental health and general wellbeing, especially during the period of transition (HM Government, 2018; National Health Service England, 2019; Ministry of Defence, 2021a). Levels of engagement and acceptance, within both the military and civilian worlds, of holistic, peer focused and delivered, interventions have accelerated in recent years, as well as a wider acceptance of the power of targeting both mind and body (Veeninga and Hafkenscheid, 2004; Hundt et al., 2015; McGill et al., 2019). By promoting, and securing, the effective transition of our military personnel we empower them to become the valuable, contributory, members of society that they have the potential to be; fully utilizing all the skills, abilities and positive characteristics of their military careers (HM Government, 2018; National Health Service England, 2019; Ministry of Defence, 2021a). To achieve this, however, requires the full collaboration, investment and coordination of organizations and charities within the sector, and the full endorsement of this ethos by the Government, Ministry of Defense, National Health Service and third sector organizations. This emerging, holistic, viewpoint has a strong synergy with the principles of Trauma-Informed Care (TIC) that are becoming more accepted and instrumental within the approach taken by the National Health Service when addressing the needs of the nation's mental health care and support (National Health Service Northern England, 2020). At the heart of TIC is a focus upon the causes of the presented malady and a move away from the previous focus on symptoms.

### Strengths and Limitations of Review

This review seeks to be pioneering and ground-breaking in it's proposal to re-assess and re-engage with a vulnerable population which has conceivably been ostracized and estranged by traditional and reductionist outlooks and values. The power and strength of this review, therefore, lies in it's desire and aspiration to remove the blinkers, throw away the rule book and begin to move the dialogue to where it needs to be; to be current, relevant, and authentic to the needs, concerns and hopes of this population. A conscious decision was made to not utilize any quality assessment tool, such as CASP, upon the identified papers and to cast as wide a net as possible, to gather and glean as much evidence of good practice and strategy, with regards to loneliness and isolation within the PTSD diagnosed veteran community, as is feasible. Only by the laying of such broad and diverse foundations can the true worth of holistic interventions be effectively gauged, and the direction of the journey forwards be charted.

That said, the review has it's limitations. Only papers written in English, from the selected sources and utilizing the chosen search terms, were examined. Therefore, any papers outside of these parameters were excluded. The chosen search terms, and sources selected from, could be viewed as subjective and biased perhaps, depending upon one's previous experience within the use of holistic, non-traditional, interventions to mitigate loneliness and social isolation within the PTSD diagnosed veteran community?

There are several limitations to the studies identified, and as a consequence, areas for further research are identified below. Global research which directly addresses loneliness and social isolation within veteran PTSD is both limited and localized. Only 2 out of 28 studies were UK-based (Lobban and Murphy, 2018, 2020), whereas 21 were US studies (Obenchain and Silver, 1991; Ragsdale et al., 1996; Jones et al., 2000; Johnson et al., 2004, 2018; Holliday et al., 2015; Azevedo et al., 2016; Beidel et al., 2016, 2017; Trahan et al., 2016; Lawrence et al., 2017, 2019; Matthieu et al., 2017; Bergen-Cico et al., 2018; Crowe et al., 2018; Cushing et al., 2018; Pezzin et al., 2018; Weiss et al., 2018; Bolman, 2019; Bauer et al., 2021), two were conducted in Israel (Bensimon et al., 2008, 2012), one in Denmark (Galsgaard and Eskelund, 2020) and two in Australia (Otter and Currie, 2004; McLaughlin and Hamilton, 2019). Endemic cultural differences exist between the UK and the other countries, extending to their respective armed forces and veteran communities, raising the need for more UK-centric veteran research to be carried out.

Service user/career involvement in the design of the identified studies was not mentioned. It is important that service user/careers are involved in the research process to ensure the design of interventions that the community can trust and invest in. Future research should embrace service user/career involvement in the design of appropriate interventions, in compliance with the National Institute for Health Research (NIHR) (Bortoli, 2021). From the 28 identified studies, 15 directly address loneliness and social isolation, *via* the conduits of promoting social engagement and functioning (Obenchain and Silver, 1991; Johnson et al., 2004, 2018; Bensimon et al., 2008, 2012; Azevedo et al., 2016; Beidel et al., 2017; Crowe et al., 2018; Pezzin et al., 2018; Weiss et al., 2018; Bauer et al., 2021) and psychosocial functioning (Holliday et al., 2015; Lawrence et al., 2017, 2019; Matthieu et al., 2017). This trend must be cultivated and developed further, in order to give consequent conclusions and recommendations more statistical power and authority.

A significant number of the studies utilized a small research cohort (Ragsdale et al., 1996; Johnson et al., 2004; Bensimon et al., 2012; Holliday et al., 2015; Matthieu et al., 2017; Bergen-Cico et al., 2018; Lobban and Murphy, 2018, 2020; Weiss et al., 2018; Lawrence et al., 2019; McLaughlin and Hamilton, 2019; Galsgaard and Eskelund, 2020) and, therefore, only have limited statistical power and moderate effect sizes. Many of the studies are novel in approach and are, therefore, potentially both logistically complex in nature and problematic to quantify regarding efficacy (Obenchain and Silver, 1991; Otter and Currie, 2004; Bensimon et al., 2008; Lobban and Murphy, 2018, 2020), as they are moving away from established research norms of medical and psychological protocol and classification. Furthermore, many are overly-representative of white males aged 30–50; albeit that the military does recruit more males than females and there is also an age criterion for service. It would be encouraging, nonetheless, to see more female veterans and people from BAME communities within research. There were only two studies which considered African American PTSD diagnosed veterans (Jones et al., 2000; Nevins et al., 2013). A number of studies also utilize self-reporting of PTSD symptomology, rather than the “gold standard”, clinically administered, assessment (Beidel et al., 2016; Cushing et al., 2018; Pezzin et al., 2018; Bolman, 2019). This perhaps compromises the reliability of the results because some people who identify as having PTSD may not actually meet the diagnostic criteria. Furthermore, few studies conducted a pre, post and 6 month follow up. This approach would have given a clearer understanding of whether the interventions were effective in the long term, and therefore follow ups should be considered in future research.

### Future Research

More dedicated consideration needs to be given to the mitigation of veteran PTSD through holistic and bespoke measures that directly address the loneliness and social isolation elements of a PTSD diagnosed veteran's symptomology; out of the 28 identified studies only 15 directly address these issues. Future research should investigate the order and combination that interventions are carried out; for example, whether being offered a holistic intervention prior to any other interventions, such as therapy or medication, empowers a veteran to engage more with PTSD focused interventions. The lived experience, and direct involvement, of PTSD diagnosed veterans should be given prominence in the process of designing effective interventions that better understand their loneliness and social isolation. Furthermore, future research should seek to examine the potential link between early years trauma and later diagnosis of PTSD within the military and veteran communities.

## Conclusion

There has been some progress in recent years in the support offered to UK veterans, with an increasing focus on their mental health and wellbeing. Significant collaboration has been made between the Ministry of Defense, HM Government, the National Health Service and the third sector charities and support groups, capturing the synergy and clarity of focus that can be obtained when diverse organizations jointly own key decisions, share research for common aspirations and are committed to wholly altruistic ideals (Bortoli, 2021). This has resulted in a more coherent, targeted and joined up service for the veteran, PTSD-diagnosed, community and has begun to address previously identified shortcomings that existed with regards to the need to identify the unique position of the veteran community and their need for bespoke support and treatment, acknowledging the role that the community itself can take within their journey forwards (Hundt et al., 2015) and increasing receptiveness amongst the parties involved to raise awareness of the true etiology of veteran PTSD symptomology (Wilson et al., 2018, 2020; McGill et al., 2019) and the role that transition, from the military to civilian life, plays in experiences of loneliness and social isolation (HM Government, 2018; National Health Service England, 2019; Ministry of Defence, 2021a). This review highlights the instrumental position of loneliness and social isolation within the lives of the PTSD-diagnosed veteran community, and the mitigating role of holistic, non-clinical, non-trauma focused, interventions.

## Data Availability Statement

The original contributions presented in the study are included in the article/Supplementary Material, further inquiries can be directed to the corresponding author/s.

## Author Contributions

RG contributed to the data analysis, was the primary author of the manuscript, and had overall responsibility. MK, JK, and GW-M contributed to the data analysis and co-authored the manuscript. DF and GO co-authored the manuscript. All authors contributed to the article and approved the submitted version.

## Funding

The United Kingdom Armed Forces Covenant Fund Trust (AFCFT) has funded a 2 year research study, under their Tackling Loneliness Program. Submission fees for this manuscript are to be paid by Northumbria University Student, Library and Academic Services.

## Conflict of Interest

The authors declare that the research was conducted in the absence of any commercial or financial relationships that could be construed as a potential conflict of interest.

## Publisher's Note

All claims expressed in this article are solely those of the authors and do not necessarily represent those of their affiliated organizations, or those of the publisher, the editors and the reviewers. Any product that may be evaluated in this article, or claim that may be made by its manufacturer, is not guaranteed or endorsed by the publisher.
