# Supplementary material for: Exploring the Role of Social Connection in Interventions With Military Veterans Diagnosed With Post-traumatic Stress Disorder: Systematic Narrative Review
Source: Front Psychol. 2022 Jul 8;13:873885. doi: 10.3389/fpsyg.2022.873885 (PMC9305387; doi:10.3389/fpsyg.2022.873885)
Supplement: Supplementary file 1 [file Table_1.pdf]

Supplementary material:

Paper Characteristics (all noted papers, if not mentioned, relate to PTSD diagnosed veterans)

Author	Aim	Sample	Method	Location	Findings related to loneliness/ Social isolation within PTSD
Azevedo *et al* [3] (2016)	To investigate the efficacy of a web-based skills building / health course.	*n* = 40	web-based skills building for rural women veterans.	USA	Addresses health disparities amongst minority veteran populations, Removes barriers to interaction.
Bauer *et al* [4] (2021)	To investigate the efficacy of a web-based skills building / health course.	*n* = 80	web-based skills building for rural male & female vets.	USA	Importance of emotional awareness and management, placing emphasis on relationship and interpersonal skills.
Beidel *et al* [5] (2016)	To explore the suitability of virtual reality exposure and group intervention.	*n* = 122	Intensive outpatient therapy with virtual exposure / group work.	USA	Social functioning and reintegration are vital factors, as is social and Emotional rehabilitation.
Beidel *et al* [6] (2017)	To explore the suitability of virtual reality exposure and group intervention.	*n* = 179 Iraq / Afghan.	Intensive outpatient therapy with virtual exposure / group work.	USA	A high priority is placed upon social reintegration.
Bensimon [7] (2008)	To investigate the suitability of drum therapy.	*n* = 9 aged 20-23	Drum therapy for chronic PTSD.	Israel	Group cohesion is a vital element. It is important to restore social relationships and to consider the loneliness v togetherness paradigm.
Bensimon [8] (2012)	To investigate the suitability of Group Music Therapy.	*n* = 9 aged 20-23	Group Music Therapy for chronic PTSD.	Israel.	Develops a sense of belonging and identity and improves interpersonal communication.
Bergen-Cico *et al* [9] (2018)	To explore the efficacy of PTSD dog ownership and bonding.	*n* = 60	PTSD dog bonding. Dogs2Vets program.	USA	Targets isolation, develops self-compassion and self-judgement.
Bolman *et al* [10] (2019)	To investigate the suitability of bonding with traumatised parrots.	not stated.	Traumatised parrot bonding for PTSD.	USA	Becoming well together through relationships of mutual care.
Crowe *et al* [18] (2018)	To investigate the suitability of PTSD dog ownership.	*n* = 9	PTSD dog ownership. Paws+Stripes program. Focus groups.	USA	Targets isolation and develops a sense of reconnection. Improves social opportunities and develops a sense of worth and purpose.
Cushing *et al* [19] (2018)	To investigate the suitability of trauma sensitive yoga.	*n* = 9 aged 22-52	Trauma sensitive yoga. for PTSD vets from Iraq/ Afghan with PCL of 30+	USA	Restores social connection and improves body awareness and wellbeing.
Galsgaard *et al* [20] (2020)	To investigate the suitability of PTSD dog ownership.	*n* = 5 aged 38-57	PTSD dog ownership for vets with active service. Care for a puppy and Group work.	Denmark	Develops a sense of commitment and responsibility through building attachment.
Holliday *et al* [24] (2015)	To explore the efficacy of Cognitive Processing Therapy.	*n* = 121	Cognitive Processing Therapy for male / female veterans with Military Sexual Trauma PTSD.	USA	Develops social functioning skills. Limited benefits only, not for overall quality of life.
Johnson *et al* [28] (2004)	To investigate the suitability of horse riding therapy.	*n* = 57 aged 18+ weight limit fitness level.	Horse riding therapy for PTSD for vets under 220 lbs weight and fit.	USA	Builds self-confidence and efficacy and develops social engagement.

Johnson et al [29] (2018)	To explore the efficacy of Intensive inpatient programme.	n = 3	15 week intensive multi-disciplinary inpatient programme for veteran and family.	USA	Maladaptive coping mechanisms decreased. Self-reporting for PTSD and self-esteem improved Friendships and family links better.
Jones *et al* [30] (2000)	To investigate the suitability of Group Therapy for the African American community.	*n* = 153	Group Therapy for African American community PTSD.	USA	Concentrates on a sense of social continuity and connectedness.
Lawrence *et al* [34] (2017)	To investigate the suitability of a Civic Service programme.	*n* = 67 TBI Iraq/ Afghan.	Civic Service programme.	USA	Builds self-purpose, focus and a valued contributing position within the group dynamic.
Lawrence *et al* [35] (2019)	To investigate the suitability of a Civic Service programme.	*n* = 346 Post 9/11	Civic Service programme for post 9/11 veterans.	USA	Builds self-purpose, focus and a valued contributing position within the group dynamic.
Lobban *et al* [37] (2018)	To explore the efficacy of an art therapy / workshop.	*n* = 4	Art therapy intervention for experiential avoidance within PTSD. Focus groups.	UK	Builds universality, cohesion, interpersonal development and development of hope. Improves day to day functioning.
Lobben *et al* [38] (2020)	To investigate the suitability of art and museum collection therapy.	*n* = 8	Art and museum collection therapy. Combat Stress. Focus groups.	UK	Developing self-confidence through increased social interactions and an improved sense of belonging.
Matthieu *et al* [40] (2017)	To investigate the suitability of a Civic Service programme.	*n* = 346 Post 9/11	Civic Service programme for post 9/11 veterans.	USA	Builds self-purpose, focus and a valued contributing position within the group dynamic.
McLaughlin *et al* [44] (2019)	To investigate the suitability of PTSD dog group therapy.	*n* = 10	PTSD dog group therapy.	Australia	Group dynamic aids engagement. Emotion regulation improved. Positive life engagement.
Nevins *et al* [51] (2013)	To investigate the suitability of horse-centric therapy.	*n* = 1	The Saratoga war Horse Project PTSD horse therapy. Single case study.	USA	Aids emotional development of post deployed veterans. Fosters creativity and communication.
Obenchain [52] (1991)	To explore the suitability of 'welcome ceremony'.	n = 3	Welcome ceremony for Vietnam veterans. Treats 'Sanctuary trauma'.	USA	Re-contextualises trauma and safe space. Improves emotional constriction and re-integration with society.
Otter *et al* [53] (2004)	To investigate the suitability of a supervised aerobic Exercise programme.	*n* = 5 Vietnam.	Supervised aerobic exercise programme for Vietnam vets. Focus groups.	Australia	Increases self-motivation, builds positive daily habits and resilience and constructs social support.
Pezzin *et al* [55] (2018)	To investigate the suitability of guitar instruction therapy.	*n* = 40	Guitar instruction therapy for PTSD.	USA	Develops social connections and increases self-esteem and resilience.
Ragsdale *et al* [59] (1996)	To investigate the suitability of inpatient adventure therapy.	*n* = 48 aged 40-49 Vietnam.	Inpatient adventure therapy for Vietnam veterans. Group work.	USA	Creation of non-judgemental, accepting environment for normalisation and management.
Trahan *et al* [75] (2016)	To explore the efficacy of a modular telephone-based therapy programme.	*n* = 1	Telephone-based modular CBT programme. Single case study.	USA	Develops social interaction skills.
Weiss *et al* [79] (2018)	To investigate the suitability of a web-based skills building programme.	*n* = 51	Web-based skills building for rural women veterans who have experienced Military Sexual trauma.	USA	Concentrates on normalisation and acceptance through social engagement.
